# Measurement of sexual health outcomes among people who inject drugs: pilot study in Catalonia, Spain and Barnaul, Russia

**DOI:** 10.1186/s12889-018-5065-x

**Published:** 2018-01-29

**Authors:** Cinta Folch, Lev Zohrabyan, Natalia Vagaitseva, Jordi Casabona, Igor Toskin, the local REDAN Group

**Affiliations:** 1 Centre d’Estudis Epidemiològics sobre les Infeccions de Transmissió Sexual i Sida de Catalunya (CEEISCAT), Departament de Salut, Generalitat de Catalunya, Badalona, Spain; 2 Department of Paediatrics, Obstetrics and Gynaecology, and Preventive Medicine, Universitat Autònoma de Barcelona, Badalona, Spain; 3 CIBER Epidemiologia y Salud Pública (CIBERESP), Madrid, Spain; 4 Joint United Nations Programme on HIV and AIDS (UNAIDS), Moscow, Russian Federation; 5 Altai Regional AIDS center, Barnaul, Russia; 6 World Health Organization, Department of Reproductive Health and Research, Geneva, Switzerland

**Keywords:** Sexual health, People who inject drugs, Surveys, Social norm, Sexual satisfaction

## Abstract

**Background:**

The main objective of this study was to test some of the draft sexual health indicators developed by the World Health Organization as part of a comprehensive indicator framework to monitor progress in universal access to reproductive health.

**Methods:**

Cross-sectional studies among people who inject drugs were conducted in Catalonia (*n* = 734) and Barnaul (*n* = 500). ‘Sexual competency’ was measured using three indicators: sexual satisfaction, sexual safety, and sexual autonomy. Individual social norms on sexuality were also collected. The construct validity of the sexual safety and sexual autonomy items were assessed. Multivariate logistic regression models explored factors associated with sexual dissatisfaction.

**Results:**

In Catalonia, sexual competency was higher among males than females who inject drugs (60.4% versus 33.3%). In both Catalonia and Barnaul, differences by sex in social norms on sexuality were seen. Mean scores on sexual safety (4.15 in Catalonia and 3.54 in Barnaul) were lower among participants who reported not using condoms. Mean scores on sexual autonomy (4.42 in Catalonia and 3.97 in Barnaul) were lower among those who had experienced some form of sexual assault. Perceived sexual safety, sexual autonomy, and social norms on sexuality were associated with sexual dissatisfaction.

**Conclusions:**

The sexual health indicators tested are valid, feasible, and reliable tools to monitor and evaluate sexual health programs and activities. The results confirm that sexual satisfaction depends on safe sexual experiences, free from coercion and violence. Social norms and individual perceptions about sexual health need to be considered when developing national disease prevention programs.

## Background

According to the World Health Organization (WHO), sexual health is a state of physical, emotional, mental, and social well-being in relation to sexuality and not merely the absence of disease, dysfunction, or infirmity. [1] Like many other areas of health, sexual and reproductive health is multidimensional, influenced by a complex interplay of biological, psychological, and social determinants. [2] A number of conventions and agreements over the past two decades, including the International Conference on Population and Development in 1994 and the Millennium Development Goals, established a strategic framework to advance sexual and reproductive health globally. More recently, sexual and reproductive health and reproductive rights have been included in the Sustainable Development Goals framework. The goals and targets encompass many key aspects of sexual and reproductive health and rights, including access to sexual and reproductive health services, comprehensive sexuality education, and the ability to make decisions about one’s own health. In this context, the WHO Department of Reproductive Health and Research (RHR), in collaboration with partners, developed a draft set of 17 sexual and reproductive health indicators as part of a comprehensive indicator framework to monitor progress towards universal access to sexual and reproductive health. [3–5]

In 2007, RHR and its technical partners developed an initial set of sexual health indicators (SHIs) ranged from process/activity (e.g. policy- and services-related) through outcome (e.g. behavioral change) to impact (e.g. prevalence of certain sexual health conditions in different populations). This initial work created a solid basis for moving forward and strengthening national and institutional capacity to monitor and evaluate outputs, outcomes, and the impact of sexual health programs. Taking into account the differences in the indicators proposed in 2007 with regard to clarity of definition, tested feasibility, and comprehensiveness, further work was done to harmonize the indicators in order to provide standard definitions and tools for measurement and reporting. The process allowed the identification of a core set of sexual health indicators (policy and laws related to sexual health, availability of and access to sexual health services and coverage, and outcome and impact indicators) that can be easily integrated into existing national and sub-national monitoring and evaluation mechanisms for routine monitoring and reporting.

Some of the proposed indicators have been submitted for validation through special surveys among men who have sex with men and people who inject drugs (PWID) in the WHO European region. The main objective of this article is to describe the results of the pilot study conducted in Catalonia (Spain) and Barnaul (Russia) to test some of the SHIs in terms of their feasibility, acceptability, and utility within the context of special surveys and cross-sectionals studies among PWID. A secondary objective was to examine the relationship between sociodemographic and behavioral factors and sexual satisfaction. The descriptive analysis of national policies and laws related to sexual health, as well as normative documents related to the availability of and/or access to sexual health services in both countries. This review was conducted in order to provide a policy snapshot of the situation in each country and to better understand the contextual factors that may play a role in sexual health.

## Methods

### Development of the core set of sexual health indicators

RHR established an international advisory group on SHIs in 2011. The main objective was to identify a set of core SHIs to be recommended for use within sexual health programs across all WHO regions.

The process began by searching for information on indicators in published and unpublished literature made available since 2007. Published sources were identified from public health databases and unpublished from technical reports requested from the appropriate points of contact at each of the WHO regional offices. A comprehensive Medical Subject Headings (MeSH)-based search strategy was developed to search the PubMed website for each sexual health topic and a special search strategy was developed for Popline. The searches were restricted to documents published in English. Sixty-one articles were found containing indicators and measurements related to sexual health (from a total of 707 publications). Among these, 24 articles containing measurement scales, inventories, and indices were included in the final database. In parallel, all six WHO regional offices and the World Association for Sexual Health (WAS) were contacted to identify the indicators used within their sexual health programs and projects.

The collated information was used to develop the first draft of the ‘Core Set of Sexual Health Indicators’ in 2012. The proposed indicators covered healthy sexuality, sexual well-being, sexual dysfunction, sexual vulnerability, sexual violence, harmful practices, and adolescent sexual health. The document recommended 15 outcome/impact and determinant indicators as well as the ‘National Commitments and Policy Instrument’ (NCPI) tool to assess the policy context and environment. WHO has reviewed the draft Core Set of Sexual Health Indicators internally and through its regional offices. Other United Nations agencies and technical partners such as The Joint United Nations Programme on HIV/AIDS (UNAIDS), United Nations Office on Drugs and Crime (UNODC), United Nations Population Fund (UNFP), United Nations Educational, Scientific and Cultural Organization (UNESCO) Chair for Sexual Health and Human Rights, WAS, and members of the WHO Gender and Rights Advisory Panel have also reviewed the above draft indicators.

### Pilot study: Bio-behavioral surveys in Catalonia and Barnaul

The Core Set of Sexual Health Indicators was piloted among PWID in Catalonia and Barnaul in 2012–2013. Cross-sectional studies were implemented to recruit a sample of 735 PWID in Catalonia [6] and 500 in Barnaul. [7]

#### Sampling and data collection (Catalonia)

In Catalonia, a convenience stratified sample of PWID attending harm reduction centers were selected during 2012–13 according to the type of center and country of origin using proportional allocation, as part of the Integrated HIV and Sexually Transmitted Infections (STI) Surveillance System in Catalonia (SIVES). These centers provide needle exchange programs, outreach programs, and supervised injecting facilities. Individuals who reported having injected in the previous 6 months and who attended these centers were eligible to take part. Those who agreed to participate signed the informed consent document and received EUR 24.

Trained interviewers conducted face-to-face interviews using an anonymous structured questionnaire. [8] The questionnaire was translated into Spanish, Romanian, Russian, English, and French, and included questions on sociodemographic characteristics, drug use and sexual behavior, knowledge of HIV and HCV status, and previous history of STI. The majority of questions regarding behavior referred to the preceding 6 months. HIV and Hepatitis C tests were taken anonymously using the ORASURE instrument (Epitope Inc., UK). Anti-HIV antibodies were detected in oral fluid using Detect-HIV version 4 from ADALTIS; [9] anti-HCV antibodies were detected using HCV 3.0 SAVE ELISA from Ortho-Clinical Diagnostics. [10] The protocol for this study received ethical approval from the Hospital Universitari Germans Trias i Pujol Ethics Committee.

#### Sampling and data collection (Barnaul)

Respondent driven sampling (RDS) methodology was used to collect bio-behavioral data among PWID in Barnaul. This is a chain referral sampling method that collects data on social network sizes and recruitment patterns to determine selection probabilities. [11]. The sample size was calculated for 500 respondents based on a confidence of 95%, power of 80% and a design effect of 2.0. Recruitment was initiated with five initial recruits identified through non-governmental organizations providing services to PWID. Once participants provided informed consent and completed the survey requirements (face-to-face interview in Russian, pre-test counselling, and venous blood draw for HIV, HCV, and syphilis testing), they received three coupons to use to recruit additional participants into the survey. All participants received about USD 15 for completing the survey process and USD 10 for each eligible recruit who participated in the survey. In addition, participants’ personal network size (the number of people who fulfil the survey eligibility criteria with whom the participant has a reciprocal relationship and has seen in the previous month) and recruitment links were collected for data analysis.

The questionnaire included items about socio-demographic characteristics, injection drug use, and sexual risk behaviors. Participants were asked about the number of people they know, who also know them, whom they have seen in the previous month and who meet the study eligibility to fulfill requirements for RDS analysis. Unique study identification numbers were used to link participants’ coupons, questionnaires, specimens, and test results and to facilitate confidentiality. A coupon manager system was used to monitor recruitment, duplicity, and incentive distribution. No personal data were collected. From each participant, 5 ml of venous blood was processed at the Altai Regional AIDS Center laboratory in accordance with the national guidelines. HIV antibodies were screened with Combitest HIV-1/2 AT/Ag (Комбитест ВИЧ 1/2 АГ/АТ) and confirmed with Genscreen ULTRA HIV Ag-Ab. Participants had the opportunity to receive their HIV test results, together with post-test counselling, 2 days after enrolment in the Altai Regional AIDS Center. Those with positive HIV test results were referred for treatment. Antibodies for Hepatitis C were screened with Diagnostic Systems ELISA anti-HCV and confirmed with Diagnostic Systems ELISA anti-HCVspectr GM. The Local Ethics Committee approved these surveys.

### National Commitments and policy instrument on sexual health

The NCPI on Sexual Health measures progress in the development and implementation of national sexuality policies, strategies, and laws. After the data collection, the study team conducted a desk review of documents in the area of policies and laws related to sexual health and indicators about availability/access to sexual health and education services. This took 2 months and was conducted in both Barnaul and Catalonia.

### Policies and laws related to sexual health indicators

1. Consensual sexual activity and age of consent: Existence of laws, regulations, and/or policies that limit consensual sexual activities in general or below a certain age.
2. Discrimination in relation to sexual health:
  - Existence of non-discrimination laws, regulations, and/or policies on grounds of age, sex, sexual orientation, gender identity, disability, race/ethnicity, marital status, HIV status, involvement in sex work, and socioeconomic status.
  - Existence of laws, regulations, and/or policies that foster equal opportunities for marginalized populations, such as women (including pregnant women), adolescents, people living with HIV/AIDS, people living in poverty, men who have sex with men, women who have sex with women, transgender people, intersex people, sex workers, migrants, indigenous populations, and people with disabilities.
3. Existence of laws, regulations, and/or policies concerning sex work.
4. Existence of laws, regulations, and/or policies prohibiting sexual violence.
5. Existence of a national law, regulation, policy, and/or strategy to prevent and respond to sexual violence and domestic violence, including intimate partner violence, child abuse, and marital rape.

### Availability/access to sexual health services

1. Provision of medical (including contraception, HIV voluntary counselling and testing, STI testing), psychological, and legal support for victims of sexual violence.
2. Provision of medical, psychological, and other relevant services for people (women, men, transgender people, and adolescents) with sexual dysfunction.

### Sexual health indicators

1. Sexual competency: Composite indicator as a proxy measure of sexual well-being through individuals’ perceptions and beliefs regarding their sexual life and experience within the context of autonomy, safety, and satisfaction.

This indicator was constructed from responses to the following set of prompted questions:

- Sexual safety: “The sex I have had was always as safe as I wanted it to be”.
- Sexual autonomy: “I have always been able to refuse sexual practice/s I don’t want”.
- Sexual satisfaction: “I have been happy with my sex life”.

Each question was measured on a five-point Likert scale: (1) strongly disagree, (2) disagree, (3) neither/not sure, (4) agree, and (5) strongly agree. For the purpose of this analysis, each individual Likert scale was combined into two categories: 1) agree or strongly agree; 2) neither/not sure, disagree or strongly disagree. The composite indicator on sexual competency was created by combining the threenew dichotomous variables. It describes the proportion of participants who agree or strongly agree to all three statements among those who reported sexual activity in the last 6–12 months: 1) agree or strongly agree to all three statements; 2) all other options.

2. Social norms on sexuality: Individual perceptions towards social norms addressing gender identities and roles, relationships, and sexual orientation.

Participants were asked to respond to the following three questions about opinions:

- One can have sex without loving the partner.
- Because of nature, men have a greater sexual need than women.
- Homosexuality is sexuality, the same as any other.

Each question was measured on a five-point Likert scale: (1) strongly disagree, (2) disagree, (3) neither/not sure, (4) agree, and (5) strongly agree. Each individual Likert scale was combined into two categories: 1) agree or strongly agree; 2) neither/not sure, disagree or strongly disagree.

#### Statistical analysis

Analyses were presented separately for Catalonia and Barnaul. Standard descriptive statistics including frequency, proportions, means, and standard deviations were reported. Proportions were compared using the Pearson chi square and the Fisher exact test. For quantitative variables, means were compared using the Student t test after verification of the equality of variances using the Levene test. The construct validity of the sexual safety and sexual autonomy items were assessed in this study. Independent sample t-tests were used to assess differences in mean sexual safety scale scores between participants who reported condom use (always) and non-use in the previous 12 months. Independent sample t-tests were also used to assess differences in mean sexual autonomy scale scores between participants who reported previous sexual assault (last 12 months) and those who did not.

Univariate and multivariate logistic regression models explored which factors could be significantly associated with a low level of sexual satisfaction, defined as those who disagree or strongly disagree with the statement: “I have been happy with my sex life”. Variables with a significance level of < 0.10 in the univariate analysis were included in the multivariate analysis, after adjusting for sex, age, and origin (in Catalonia), and the Odds Ratio (OR) with its respective 95% confidence interval (CI) were calculated. Final multivariate models were derived using a backward elimination process. Statistical significance was set at *P* < 0.05. The analyses were performed using SPSS version 17.0.

## Results

### National Commitments and policy instrument on sexual health in Barnaul and Catalonia

Table 1 presents a summary of the main policies and laws related to sexual health and indicators about availability/access to sexual health and education services in Barnaul and Catalonia.

**Table 1:** Policies and laws related to sexual health and availability/access to sexual health and education services in Catalonia and Barnaul

	Catalonia	Barnaul
Legal age for marriage without consent^1,14,26^	16 years	18 years; 16 years for partners of the same age
Same-sex marriage^2^	Legal	Not legal
% of national population under laws, regulations, and/or policies supporting non-discrimination on grounds of age, sex, sexual orientation, gender identity, disability, race/ethnicity, marital status, HIV status, involvement in sex work, and socioeconomic status^3,15, 16, 17, 18, 19, 20, 21, 22, 23, 24, 25^	100%	100%
% of national population under laws, regulations, and/or policies supporting non-discrimination on grounds of:		
Women^4,15, 16, 17, 18, 19, 20, 21, 22^	100%	100%
Adolescents^5,15, 16, 17, 18, 19, 20, 21^	100%	100%
People living with HIV/AIDS^6,15, 16, 17, 18, 19, 20, 21, 23^	100%	100%
The poor^3,15, 16, 17, 18, 19, 20, 21^	100%	100%
Men who have sex with men^7,15, 16, 17, 18, 19, 20, 21^	100%	100%
Women who have sex with women^7,15, 16, 17, 18, 19, 20, 21^	100%	100%
Transgender^7,15, 16, 17, 18, 19, 20, 21^	100%	100%
Intersex people^7,15, 16, 17, 18, 19, 20, 21^	100%	100%
Migrants, indigenous populations^8,9,15, 16, 17, 18, 19, 20, 21, 24^	100%	100%
Sex workers^3,15, 16, 17, 18, 19, 20, 21^	100%	100%
People with disabilities^3,15, 16, 17, 18, 19, 20, 21, 25^	100%	100%
Regulation of sex work	Prostitution itself is not regulated, but exploitation such as pimping is illegal	Not regulated by the Government
% of national population under formal laws, regulations, and/or policies prohibiting sexual violence^10,14,27, 28, 29, 30 , 31, 32, 33, 34, 35, 36, 37, 38, 39, 40^	100%	100%
Existence of a national law, regulation, policy, and/or strategy to prevent and respond to sexual violence and domestic violence, including intimate partner violence, child abuse, and marital rape^11,12,27, 28, 29, 30, 31, 32, 33, 34, 35, 36, 37, 38, 39, 40^	Yes	Yes
Provision of medical, psychological, and legal support for victims of sexual violence^13,27,28,29,30^	Yes	Yes
Support services for people with sexual dysfunction	Attention to sexual and reproductive health units at primary health care centres	Not regulated

^1^http://www.congreso.es/public_oficiales/L10/CONG/BOCG/A/BOCG-10-A-112-1.PDF; ^2^Law 13/2005 that amends the Civil Code regarding the right to contract marriage; ^3^ Spanish Constitution; article 14 [Equality]; ^4^Organic Act 3/2007 of 22 March for effective equality between women and men; ^5^Law 14/2010 of May 27, the rights and opportunities in childhood and adolescent; ^6^National Agreement to deal with the HIV related stigma and approved on March 6, 2014 the Parliament of Catalonia; ^7^Law 11/2014, of 10 October, to guarantee the rights of lesbian, gay, bisexual, transgender, and intersex people and to eradicate homophobia, biphobia, and transphobia (only in Catalonia); ^8^Act 10/2010 of the 7th May, on reception for immigrants and returnees to Catalonia; ^9^In Catalonia, as provided in Instruction 10/2012 of 30 August 2012 of the Government of Catalonia / CatSalut, not regularized immigrants continue to have access to the health system through a one-year renewable permit, which can be applied for if credited 3 months of continued registration in Catalonia and having an income below the Basic Income for Inclusion and Social Protection; ^10^Organic Act 1/2004 of 28 December on Integrated Protection Measures against Gender Violence; ^11^ Organic Law 10/1995, of 23 November, Criminal Code; ^12^Law 5/2008 on the right of women to eradicate sexist violence; ^13^Protocol for Dealing with Sexist Violence in the Healthcare Field in Cataloniahttp://salutweb.gencat.cat/web/.content/home/ambits_tematics/linies_dactuacio/model_assistencial/ordenacio_cartera_i_serveis_sanitaris/abordatge_de_la_violencia_masclista/documents/arxius/eng_femchist.pdf); ^14^Article 134 of the Criminal Code of the Russian Federation; ^15^Section 2, articles 17-64 of the Constitution of the Russian Federation; ^16^Article 5.62 of the Administrative Offences Code of the Russian Federation dated by 30.12.2001; ^17^ Federal Law № 195 (new version of the law dated by 21.07.2014); ^18^ Article 3 of the Labor Code of the Russian Federation; ^19^ Article 136 of the Criminal Code of the Russian Federation; ^20^ Federal Law № 420 (new version of the law dated 07.12.2011); ^21^Article E of the European social charter ratified by the Federal Law № 101 on 03.06.2009; ^22^Article 52 of the Federal Law № 323 dated 21.11.2011 (new version of the law dated 21.07.2014); ^23^ Article 5 of the Federal Law № 38 dated 30.03.1995 (new version of the law dated 28.12.2013 with the amendments dated 04.06.2014); ^24^ Article 3 and 4 of the Federal Law № 115 dated 25.07.2002 (new version of the law dated 21.07.2014); ^25^Federal Law № 181 dated 24.11.1995; ^26^ Article 13 of the Family Code of the Russian Federation; ^27^ Criminal Code of the Russian Federation; ^28^ Federal Law № 323 “On fundamental healthcare principles in the Russian Federation” dated 21.11.2011 (new version of the law dated 21.07.2014), articles 1-101; ^29^ Regulations of the Ministry of Labor of the Russian Federation: Order No 681n of the Ministry of Labor dated 18.11.2013; ^30^ Regulations of the Government of the Russian Federation: Decree No 995 of the Government of the Russian Federation dated 06.11.2013; ^31^Article 131 of the Criminal Code of the Russian Federation; ^32^ Federal Law № 63 dated 13.06.1996(new version of the law dated 21.07.2014); ^33^Article 132 of the Criminal Code of the Russian Federation; ^34^ Federal Law № 215 dated 27.07.2009; ^35^ Article 133 of the Criminal Code of the Russian Federation; ^36^ Federal Law № 420 dated 07.12.2011; ^37^ Federal Law № 14 dated 29.02.2012; ^38^ Article 135 of the Criminal Code of the Russian Federation; ^39^ Article 127 of the Criminal Code of the Russian Federation, section 17: Crimes against life, liberty, and dignity of person; ^40^ Crimes against health: articles 111-125 of the Criminal Code of the Russian Federation

This review found considerable similarities between Catalonia and Barnaul in terms of the existence of national policies, strategies, and laws related to sexual health at population level. Nevertheless, same-sex marriage has been legal in Spain since July 2005, while neither same-sex marriage nor civil unions of same-sex couples are allowed in Barnaul. In addition, there are no specific laws and regulations addressing the needs of people with sexual dysfunction in Russia, but the services of andrologists and gynecologists are provided by hospitals and out-patient facilities across the country in the framework of primary and secondary medical care.

### Sample characteristics

A total of 735 PWID completed the survey in Catalonia and 500 in Barnaul. Table 2 summarizes the main socio-demographic and behavioral characteristics of the samples.

**Table 2:** Socio-demographic and behavioural characteristics of the samples (%)

	Catalonia (*n* = 735)	Barnaul (*n* = 500)
Age (mean, SD)	38.0 (8.1)	32.3 (7.2)
Sex: Male	82.9	69.0
Migrants	39.5	NA
University studies	7.3	8.6
Employed	13.2	29.5
Years of injection (mean, SD)	15.8 (9.9)	12.8 (6.9)
History of incarceration	68.0	40.6
Last injection: last month	91.6	58.4
Sexual activity*	82.3	94.2
Number of partners**		
One	82.9	69.0
Two	48.6	46.3
Three or more	20.6	15.5
IDU sex partner	30.8	30.8
Sex work***	20.3	51.8
Sexual violence*	5.0	1.7
Condom (last sex)	2.6	3.8
Condom (last sex with steady partner)	NA	27.8
Condom (last sex with casual partner)	38.8	NA
Always using condoms*	74.6	NA

*last 12 months; ** among those who reported sexual activity in the last 12 months; **last 6 and 12 months in Catalonia and Barnaul, respectively; *NA* not available

### Sexual health indicators

#### Sexual competency

Table 3 presents the distribution of participants’ views on different items measuring sexual competency: sexual safety, sexual autonomy, and sexual satisfaction. In Catalonia, there were differences among male and female PWID in all three items, whereas in Barnaul the differences were almost significant for the sexual safety and satisfaction items.

**Table 3:** Proportion of participants who agree/strongly agree with the specific items on sexual competence indicator

	Catalonia^a^			*p*	Barnaul^b^			*p*
	Global *n* = 549	Male *n* = 444	Female *n* = 105		Global *n* = 471	Male *n* = 325	Female *n* = 146	
Protection self-efficacy^1^	77.5	82.0	58.1	< 0.001	53.4	56.0	47.3	0.079
Consensual sex^2^	86.8	90.3	71.4	< 0.001	79.6	80.3	78.1	0.579
Satisfaction^3^	72.8	75.7	60.0	< 0.001	63.0	65.5	56.8	0.071

^a^participants who reported sexual activity in the last 6 months; ^b^participants who reported sexual activity in the last 12 months; 1- The sex I have had was always as safe as I want it to be; 2- I have always been able to refuse sexual practice/s I don’t want; 3- I have been happy with my sex life

**Fig. 1:**
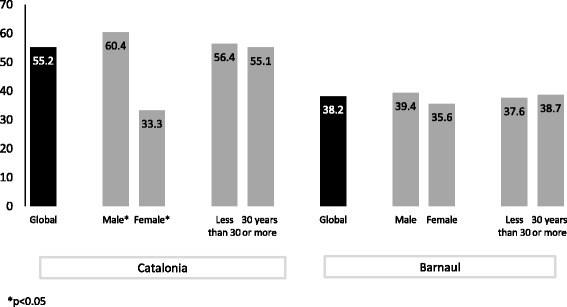
Composite indicator on sexual competency by age and sex

When the three items were combined (composite indicator), differences by sex were seen in Catalonia: sexual competency was higher among male than female PWID (60.4% vs. 33.3%). No differences by age and sex were seen in the composite indicator in Barnaul. Globally, the sexual competence indicator was higher among participants from Catalonia than from Barnaul (55.2% and 38.2%, respectively, *p* < 0.005) (Fig. 1).

#### Social norms on sexuality

Table 4 presents the distribution of participants according to the different items measuring individual perceptions towards social norms addressing gender identities and roles, relationships, and sexual orientation. A higher proportion of males than females agreed with the statement: “one can have sex without loving the partner” in both Catalonia and Barnaul. Differences by sex were seen also in Catalonia in the proportion who agreed with the statement: “Because of nature, men have a greater sexual need than women” (56.0% and 34.2% of female and male, respectively). On the other hand, negative attitudes towards non-traditional sexual orientation were higher among male than female PWID in both Catalonia and Barnaul, with a greater proportion of men than women agreeing with the statement: “homosexuality is sexuality, the same as any other”.

**Table 4:** Proportion of participants who agree/strongly agree with the specific items on social norms

	Catalonia			Barnaul		
	Global *n* = 731	Male *n* = 606	Female *n* = 125	Global n = 471	Male *n* = 326	Female *n* = 145
One can have sex without loving the partner	70.6	72.8^a^	60.0^a^	69.9	73.3^a^	62.1^a^
Because of nature, men have more sexual needs than women	38.1	34.2^a^	56.0^a^	60.3	61.0	58.6
Homosexuality is a sexuality as any other	66.6	63.3^a^	81.6^a^	7.4	3.4^a^	16.6^a^

^a^Significant differences by sex

### Construct validity on protection self-efficacy and consensual sex

The mean scores on the sexual safety scale were 4.15 (1.26) and 3.54 (1.04) in Catalonia and Barnaul, respectively. Participants who reported using condoms in the previous 12 months had significantly higher protection self-efficacy scores than participants who reported not using condoms during this period (Table 5).

**Table 5:** Assessment of construct validity through independent sample t-tests for differences between mean scores on protection self-efficacy and consensual

Item	Test group	Mean (SD)	*p*	Mean (SD)	*p*
Protection self-efficacy scale	Condom use: always	4.61 (0.82)	< 0.001	4.11 (0.79)	< 0.001
	Condom use: not always	3.85 (1.42)		3.43 (1.05)	
Consensual sex scale	Previous sexual assault	3.53 (1.59)	< 0.001	3.47 (1.22)	0.015
	No previous sexual assault	4.45 (1.02)		4.00 (0.90)	

The mean scores on the sexual autonomy scale were 4.42 (1.06) and 3.97 (0.92) in Catalonia and Barnaul, respectively. Mean scores were lower among participants who had experienced some form of sexual assault in the previous year than those who did not (Table 5).

### Factors associated with a low level of sexual satisfaction (or sexual dissatisfaction)

At multivariate level, reporting low levels of perceived sexual safety was associated with sexual dissatisfaction in both Catalonia and Barnaul studies (OR = 1.75; 95% CI: 1.10–2.78 and OR = 3.42; 95% CI: 2.25–5.22, respectively). Furthermore, a low perception of sexual autonomy was associated with a sexual dissatisfaction (OR = 1.77; 95% CI: 1.02–3.08 and OR = 3.43; 95% CI: 2.07–5.69 in Catalonia and Barnaul, respectively). In Catalonia, female PWID (OR = 1.67; 95% CI: 1.03–2.71) had a higher probability of reporting low levels of sexual satisfaction than males. On the other hand, one of the items measuring social norms on sexuality was associated with sexual dissatisfaction in the Barnaul sample: participants who disagree with the statement: “Because of nature, men have a greater sexual need than women” reported low levels of sexual satisfaction (OR = 1.93; 95% CI: 1.27–2.94) (Table 6).

**Table 6:** Assessment of construct validity through independent sample t-tests for differences between mean scores on protection self-efficacy and consensual sex

	aOR	95%IC	*p*	aOR	95%IC	*p*
Sex						
Male	1.00			1.00		
Female	2.56	1.51–4.32	< 0.0001	1.31	0.84–2.04	0.227
Age group						
Less than 30	1.00			1.00		
30 or more	1.81	1,06–3,09	0,029	1.12	0.73–1.70	0.607
Perception of sexual safety						
High	1.00			1.00		
Low	2.09	1,29–3,38	0.003	3.42	2.25–5.22	< 0.0001
Perception of sexual autonomy						
High	1.00			1.00		
Low	1.88	1.06–3.32	0.030	3.43	2.07–5.69	< 0.0001
Men more needs than women						
Agree/totally agree	_			1.00		
Disagree/totally disagree	_			1.93	1.27–2.94	0.002

### Item analysis (acceptability)

The proportion of missing data for the items was very low, with a mean proportion of missing values of 1% in both Catalonia and Barnaul.

## Discussion

The purpose of this study was to test a comprehensive draft set of SHIs (developed by WHO), as part of a comprehensive indicator framework to monitor progress in universal access to reproductive health. The findings suggest that the indicators are valid, feasible, and reliable and can be used as a tool to assess sexual health in integrated bio-behavioral surveys. Moreover, the items were highly acceptable, as evidenced by the low average rate of missing data (1%), suggesting that the participants were willing to answer sensitive questions on sexual experiences.

The sexual competence indicator piloted in this study was designed to capture perceptions and beliefs regarding sexual life and experiences within the context of autonomy, safety, and satisfaction. Several relationships explored in the data supported the construct validity of the items on protection self-efficacy and consensual sex scales. This suggests the indicator can be useful as a proxy measure of a level of sexual well-being in a survey population.

The results of the study support one of the draft criteria for healthy sexuality, in which sex life satisfaction depends on having safe sexual experiences, free from coercion and violence. [1] Previous studies have suggested an association sexual satisfaction with classic measures of sexual health as well as with mental health. [12, 13] The results presented here confirm those of previous studies in which patterns of sexual satisfaction are shaped by the interaction of individual factors and the social and cultural environment. [14, 15] Adopting a sexual health framework to enhance traditional interventions could ensure that a greater variety of important factors in sexual behaviors are addressed more comprehensively and efficiently. [16] It is therefore essential that the notions of ‘well-being’ and a ‘positive approach’ guide the construction of any new sexual health framework. In addition, when developing public health responses, a syndemic approach to prevention that considers connections between a variety of health-related problems would strengthen the design of these frameworks. [17]

These results show that lack of sexual satisfaction is more common in women than men, and this is consistent with previous studies. [18] Barnaul has the highest proportion of people who expressed traditional attitudes concerning gender and sexuality. People who deviated from these norms could perceive themselves to be socially excluded; in fact, in Barnaul, non-conformity with traditional social norms governing gender and sexuality was found to be related to reduced sexual satisfaction.

This finding is consistent with studies showing an increase of negative emotions when they deviate peer descriptive norms. [19] Other findings support the hypothesis that perceptions of descriptive social norms do indeed play a role in general sexual satisfaction, with perceived deviation from the norm leading to decreased satisfaction ratings. [20] The interplay of local legislative contexts, social norms, and individual perceptions should be considered in any sexual health intervention. In this sense, some differences between Russia and Catalonia in terms of policies and laws related to sexual health have emerged from the desk review, although a comprehensive policy content analysis should be done to better understand the current country situation, provide examples of good practice for others to learn from, and to identify gaps for further improvement.

This study has some limitations. Firstly, the study was conducted in a convenience sample of PWID attending harm reduction centers in Catalonia and so it may not be possible to generalize the results to other populations of PWID. Secondly, although the Barnaul sample was recruited through RDS methodology, the data were not weighted to control for differences in network size and clustering across groups. Any comparison between the Barnaul and Catalonia samples should therefore be undertaken with caution. Furthermore, even though self-reported risk behaviors have been found to be valid and are not influenced by social desirability bias, some risk behaviors may have been underestimated. [21] On the other hand, cross-sectional surveys cannot determine causality, complicating the interpretation of associations. In addition, SHIs have been piloted in a specific population of PWID, thus results may not be mirrored in the general population. Finally, this pilot study did not perform a qualitative assessment of the indicators and further study is needed to determine their clarity and comprehensibility.

## Conclusion

In conclusion, the composite draft SHI developed by WHO seems feasible and appropriate, both as a tool for future research and for monitoring and evaluation of sexual health-related programs and/or activities. Social norms and especially individual perceptions about sexual health need to be considered when developing national HIV/STI prevention programs.

## Abbreviations

NCPI: National Commitments and Policy Instrument
PWID: People who inject drugs
RDS: Respondent driven sampling
RHR: Department of Reproductive Health and Research
SHI: Sexual health indicators
STI: Sexually Transmitted Infections
UNAIDS: The Joint United Nations Programme on HIV/AIDS
UNESCO: United Nations Educational, Scientific and Cultural Organization
UNFP: United Nations Office on Drugs and Crime (UNODC), United Nations Population Fund
WAS: World Association for Sexual Health
WHO: World Health Organization
